# Roles of Sphingolipid Metabolism in Pancreatic β Cell Dysfunction Induced by Lipotoxicity

**DOI:** 10.3390/jcm3020646

**Published:** 2014-06-20

**Authors:** Julien Véret, Lara Bellini, Paola Giussani, Carl Ng, Christophe Magnan, Hervé Le Stunff

**Affiliations:** 1 University Paris Diderot, Sorbonne Paris City, Unit of Functional and Adaptative Biology UMR 8251 CNRS, 75205 Paris Cedex 13, France; E-Mails: julienveret@free.fr (J.V.); lbellini88@gmail.com (L.B.); christophe.magnan@univ-paris-diderot.fr (C.M.); 2 Department of Medical Biotechnology and Translational Medicine, University of Milan, LITA Segrate, Via Fratelli Cervi 93, 20090 Segrate (MI), Italy; E-Mail: paola.giussani@unimi.it; 3 School of Biology and Environmental Science and UCD Earth Institute, University College Dublin, Belfield, Dublin 4, Ireland; E-Mail: carl.ng@ucd.ie

**Keywords:** obesity, type 2 diabetes, gluco-lipotoxicity, islet of Langherans, ceramide, sphingosine-1-phosphate, sphingolipids, apoptosis, insulin, pancreas

## Abstract

Pancreatic β cells secrete insulin in order to maintain glucose homeostasis. However, various environmental stresses such as obesity have been shown to induce loss of secretory responsiveness in pancreatic β cells and pancreatic β cell apoptosis which can favor the development of type 2 diabetes (T2D). Indeed, elevated levels of free fatty acids (FFAs) have been shown to induce β cell apoptosis. Importantly, the chronic adverse effects of FFAs on β cell function and viability are potentiated in the presence of hyperglycaemia, a phenomenon that has been termed gluco-lipotoxicity. The molecular mechanisms underlying the pathogenesis of gluco-lipotoxicity in pancreatic β cells are not completely understood. Recent studies have shown that sphingolipid metabolism plays a key role in gluco-lipotoxicity induced apoptosis and loss of function of pancreatic β cells. The present review focuses on how the two main sphingolipid mediators, ceramides and sphingoid base-1-phosphates, regulate the deleterious effects of gluco-lipotoxicity on pancreatic β cells. The review highlights the role of a sphingolipid biostat on the dysregulation of β cell fate and function induced by gluco-lipotoxicity, offering the possibility of new therapeutic targets to prevent the onset of T2D.

## 1. Introduction

In mammals, the levels of plasma glucose are tightly regulated to maintain normo-glycemia, and chronic hypoglycemia or hyperglycemia can result in injuries to various tissues. Insulin secreted by pancreatic β cells plays a major role in the maintenance of normo-glycemia. The biological machinery of pancreatic β cells enables them to regulate the rate of insulin secretion in response to variations in plasma glucose levels. Diabetes is a disease characterized by a chronic abnormal elevation of plasma glucose concentrations. Type 1 diabetes is an autoimmune disease against pancreatic β cells whereas type two diabetes (T2D) is a multi-factorial disease due to two principal dysfunctions: (1) a loss of insulin secretion and (2) insulin resistance, which is defined by the failure of insulin to elicit anabolic effects and stimulate glucose uptake in targeted tissues.

The etiology of T2D is not well established but it is certain that loss of insulin secretion is directly linked to a loss of function and apoptosis of pancreatic β cells [1,2]. The insulin secretory capacity and reduction in β cell mass, observed during T2D, are thought to be amplified by the development of chronic hyperglycaemia, a phenomenon that has been termed “glucotoxicity” [2]. In addition to hyperglycaemia, accumulated evidence suggests that T2D is often associated with abnormalities in lipid metabolism and excessive levels of circulating lipids [3,4]. Indeed, it has been shown that 44% of diabetic patients are also obese [5], suggesting a link between obesity and T2D. Free fatty acids (FFAs) are important physiological fuels for islets, and act as supplemental nutrients able to potentiate insulin secretion in response to glucose [6,7]. However, chronically elevated levels of FFAs in circulation have been postulated to cause peripheral insulin resistance and impairment of β-cell insulin secretion, a phenomenon that has been termed “lipotoxicity” [8,9]. The molecular mechanisms underlying the pathogenesis of lipotoxicity in pancreatic β cells are not completely understood. Usually, low levels of FFAs are readily degraded by β-oxidation and are therefore non-toxic for β-cells. In contrast, chronic elevation of FFA levels raised the levels of long chain acyl-CoA (LC-CoA), which serve for complex lipid synthesis [8,10]. Interestingly, it has been proposed that a specific class of lipids, namely sphingolipids, and in particular ceramides, are important mediators of FFA-induced β cell dysfunction and apoptosis [8]. In the present review, we will illustrate the mechanisms involved in pancreatic β cell lipotoxicity with a special focus on the role of ceramide metabolism at the molecular, cellular and systems levels. In addition, we will also discuss the role of other sphingolipid metabolites, such as sphingosine-1-phosphate (S1P) in the control of pancreatic β cell lipotoxicity. A better understanding of the role of sphingolipid metabolism involved in pancreatic β cell lipotoxicity may open up the potential for identification of pharmacological targets for the prevention and/or treatment of obesity associated T2D.

## 2. Lipid Metabolism in Pancreatic β Cells

Lipids have many biological functions; they serve as energy reserves, signalling molecules, and as major membrane components. In pancreatic β cells, lipids are extracted from plasma lipoproteins through lipoprotein lipases secreted by pancreatic β cells [11]. Lipids are converted into long chain acyl-CoA (LC-CoA) by the Acyl-CoA synthase in the cells [12]. LC-CoA may be then exported to mitochondria through carnitine palmitoyl-CoA transferase 1 (CPT-1) before being channeled into the β-oxidation pathway in order to provide energy. Interestingly, the fate of LC-CoA in pancreatic β cells is determined by intracellular glucose levels. At low glucose concentrations, FFAs are used to produce energy through the β-oxidation pathway [13]. In contrast, at high glucose concentrations, FFA metabolism will be preferentially oriented to lipid esterification. The main metabolites able to control the lipid metabolism in response to glucose appear to be malonyl-CoA and the LC-CoA [10]. High glucose concentrations accelerate its metabolism and increase intra-cytoplasmic citrate concentration through the Krebs cycle. The resulting citrate is then converted into malonyl-CoA by the acetyl-CoA carboxylase (ACC) [14,15,16]. Normally, malonyl-CoA is used as a substrate by FFA synthase (FAS) to produce FFAs; however in pancreatic β cells, the activity of FAS is lower than the activity of ACC [17], favouring the accumulation of malonyl-CoA. Interestingly, increased malonyl-CoA levels can inhibit CPT-1 activity leading to a reduction in mitochondrial transport of LC-CoA and its subsequent β-oxidation. The consequences of cytoplasmic FFA accumulation are the production of lipids derived by various metabolic pathways, such as esterification or diglyceride synthesis [14,18], or phospholipid synthesis [19,20]. In addition to these effects, the glucose also induces the expression of genes involved in lipogenesis via the transcriptional factor SREBP1c, which activates *de novo* lipogenic genes [21].

## 3. The Phenomenon of Pancreatic β Cell Gluco-Lipotoxicity

It has been estimated by the World Health Organization that obesity is the main epidemic of the century, affecting about 1 billion people around the world [5]. Obesity has many deleterious physiological effects like cellular energy imbalances that can result in impairment of insulin secretion and the development of insulin resistance. Many hypotheses suggest a link between insulin resistance and obesity and T2D. Among these hypotheses, it has been proposed that hypoxia, fibrosis, and inflammation observed during obesity will impair adipose expansion and therefore the storage of excess FFAs. This phenomenon leads to ectopic storage of lipids [22,23,24]. The FFAs which accumulate in non-adipose tissues such as muscle and liver induce the activation of non-oxidative metabolic pathways. These pathways can lead to the production of toxic lipids. This phenomenon is called lipotoxicity [22,23,24]. It has been shown that lipotoxicity can result in insulin resistance of skeletal muscle, hepatic steatosis [25,26] and impairment of insulin secretion by β-cells [8,9]. Indeed, palmitate, one of the most abundant FFAs in plasma, has detrimental effects on β-cell function, including impairment of glucose-induced insulin release [27,28], defective insulin gene expression [29,30,31], and induction of β-cell apoptosis [32,33,34,35,36]. Importantly, the chronic adverse effects of FFAs on β cell function and viability are potentiated by hyperglycaemia, a phenomenon that has been termed “gluco-lipotoxicity” [37,38]. It has been observed that β cell apoptosis induced by palmitate is highly potentiated in the presence of elevated glucose concentrations [32,39]. As proposed above, when glucose and lipid levels are simultaneously elevated, glucose inhibits FFA oxidation and stimulates the incorporation not only of endogenous LC-CoA but also excess FFAs into the synthesis of complex lipids [8,10,39]. Interestingly, among these lipids produced, ceramides have been suggested to be important mediators of FFA-induced β-cell dysfunction and apoptosis [8,39,40].

## 4. Sphingolipid Metabolism

**Figure 1:**
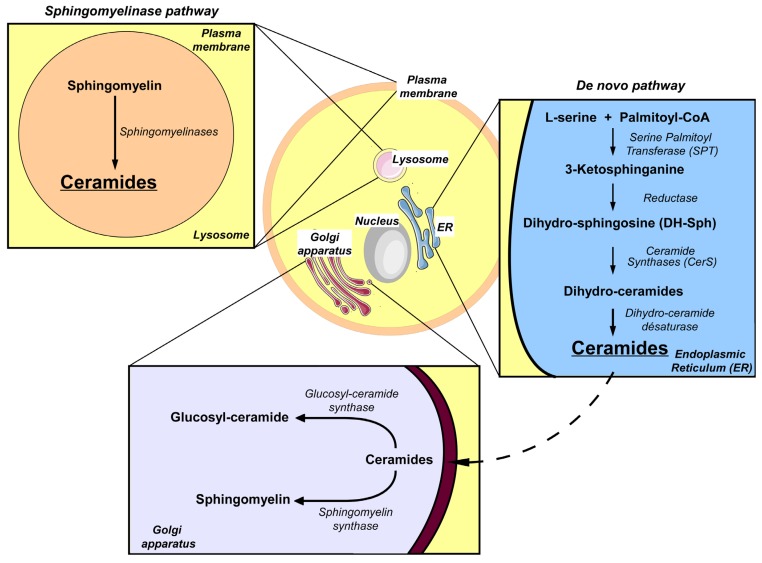
Synthesis of sphingolipids in mammalian cells. Two main pathways to produce sphingolipids exist in mammals. The *de novo* pathway of synthesis starts on the cytoplasmic face of the endoplasmic reticulum (ER). The first step of this pathway is the condensation of l-serine with palmitoyl-CoA to form a 3-ketosphinganine. This reaction is catalyzed by serine palmitoyl-transferase (SPT). 3-Ketosphinganine is then reduced to form dihydrosphingosine (DH-Sph) by 3-ketopshinganine reductase. DH-Sph is a substrate of ceramide synthases (CerS) which produce dihydro-ceramide. Dihydroceramides are transformed into ceramides by dihydroceramide desaturase. Ceramide can be transported to the Golgi apparatus to be transformed into more complex sphingolipids such as sphingomyelin and glucosyl-ceramides. A second synthesis pathway is the catabolic pathway that leads to the degradation of sphingomyelin (SM) into ceramides by sphingomyelinases. This process takes place in the lysosomal and plasma membranes.

It is well established that sphingolipids are structural components of cellular membranes. However, several studies have now shown that sphingolipids have important roles in the regulation of some cellular processes [41,42]. Moreover, the deregulation of these processes plays a key role in the onset of pathologies affecting cellular proliferation and apoptosis [42,43,44]. Several pathways for sphingolipid metabolism have been described [45]. One of these pathways is the *de novo* synthesis pathway which is initiated on the cytoplasmic face of the endoplasmic reticulum (ER) (Figure 1). The first step of this pathway is the condensation of l-serine with palmitoyl-CoA to form 3-ketosphinganine. This reaction is catalyzed by serine palmitoyl-transferase (SPT) [41]. 3-Ketosphinganine is then reduced to dihydrosphingosine (DH-Sph) by 3-ketosphinganine reductase. The resulting DH-Sph acts as a substrate for ceramide synthases (CerS) leading to the production of dihydro-ceramides. Dihydro-ceramides can be transformed into ceramides by dihydroceramide desaturases [46]. Depending on the LC-CoA attached to the DH-Sph by CerS, the dihydroceramides and the ceramides are classified into different species with varying FA chain lengths (14–32 carbons in mammals) and saturation of the carbon chain [47]. Ceramides can be transported to the Golgi apparatus [48] where they are converted into sphingomyelin by sphingomyelin synthase or into glucosyl-ceramides by glucosyl-ceramide synthase. In addition to *de novo* synthesis, another metabolic pathway also leads to ceramide production and this involves the degradation of sphingomyelin into ceramide by sphingomyelinases. This process takes place in the lysosomal membrane and the cytoplasmic membrane [41]. In this web of sphingolipid metabolism, ceramides are considered as central players in the metabolism of sphingolipids. Indeed, ceramides are the precursors of many lipid messengers. For example, ceramides can be metabolized into sphingosine which is in turn phosphorylated to yield sphingosine-1-phosphate (S1P) [41]. Ceramides can also be phosphorylated by ceramide kinase to form ceramide-1-phosphate [41].

## 5. Ceramide and Pancreatic β Cell Gluco-Lipotoxicity

As noted above, among the lipids produced during lipotoxicity, ceramides have been implicated in the deleterious effects of gluco-lipotoxicity on pancreatic β cells and in a more general manner, on T2D and obesity. For example, patients with T2D present an increase in sphingolipid levels in plasma, muscle, adipose tissue and liver [25,49]. The accumulation of sphingolipids has been correlated with an increase in insulin resistance [50,51], loss of function and death of pancreatic β cells [8]. Additionally, sphingolipids also appear to also play a role in the complication of obesity and T2D. Interestingly, the two forms of SPT (SPT 1 and 2) have a Km for l-serine and the palmitoyl-CoA near to their physiological concentrations [52,53], and as such, are sensitive to relatively small variations in the concentrations of their substrates. Moreover, condensation of l-serine and palmitoyl-coA is the rate-limiting step in *de novo* synthesis of ceramides [54]. These two characteristics of SPTs suggest that excessive concentrations of palmitate and its activated form palmitoyl-CoA in a cell can induce the aberrant production of ceramides. It has been shown that diminution of pancreatic β cell mass and loss of insulin secretion are correlated to an increase in intracellular ceramide levels and SPT expression in islet of Langerhans of obese Zucker diabetic fatty (ZDF) rats [35]. 

### 5.1. Effect of Ceramide on Pancreatic β Cell Function

Previous studies have demonstrated that cellular permeable analogues of ceramide impaired insulin production in pancreatic β-cells [55]. Under gluco-lipotoxic conditions, several studies showed a correlation between *de novo* ceramide synthesis and defective insulin expression in islet of Langerhans [8]. In islet of Langerhans and pancreatic β cell lines, ceramide dependent inhibition of insulin expression is mediated by the dephosphorylation of protein kinases, ERK1/2 by PP2A [56,57]. Moreover, the *de novo* ceramide pathway inhibits the nuclear translocation of two important transcription factors, PDX-1 and Mafa, for insulin-induced gene expression [8]. However, the intracellular signaling pathways mediating these effects are mostly unknown. Previous studies have shown that glucose regulates insulin-induced gene transcription via the serine/threonine Per-Arnt-Sim domain-containing kinase (PASK) [56]. Interestingly, palmitate decrease glucose-induced expression of PASK mRNA and protein levels [56], suggesting that ceramides may function as negative regulators of PASK. It appears that in pancreatic β cells, inhibition of ceramidase, which increased intracellular levels of ceramides, also leads to a reduction in insulin expression [57]. More recently, genetic deletion of sphingomyelin synthase 1 (SMS1) has been shown to induce accumulation of ceramides in islet of Langerhans, dysfunction of mitochondria and the subsequent inhibition of insulin secretion [58]. In Yano’s study, accumulation of ceramides affected mitochondria membrane integrity and induced an excessive production of ROS which were partially responsible for the defect in insulin secretion in pancreatic β cells [58]. Pharmacological inhibition of SMS or down-regulation by siRNA of the ceramide transporter (CERT), which regulate ceramide transport between the ER and Golgi apparatus, increased dysfunction of pancreatic β cells induced by palmitate by affecting binding of transcription factors to the insulin promoter [59]. Altogether, these results suggest that ceramide accumulation, either through the induction of *de novo* synthesis or reduced conversion into more complex sphingolipids, is involved in loss of function in pancreatic β cells.

### 5.2. Effect of Ceramide on Pancreatic β Cell Survival

It has been shown that ceramide analogues can block proliferation and induce apoptosis of β cells and islets of Langerhans in culture [60,61]. *De novo* ceramide synthesis induced by palmitate has also been implicated in pancreatic β cell apoptosis [8,39]. Indeed, inhibition of *de novo* ceramide synthesis abrogates pancreatic β cell apoptosis induced by palmitate [36,39,62]. More importantly, treatment of ZDF rats with a potent inhibitor of SPT, cyclo-serine, attenuated islet of Langerhans apoptosis and prevented the appearance of hyperglycemia [35]. Ceramide accumulation induced by (gluco)-lipotoxicity induces pancreatic β cell apoptosis through different pathways. FFAs have been shown to induce accumulation of ceramides in ER of pancreatic β cells [40,63]. To date, the mechanisms used by (gluco)-lipotoxicity to block the transport of ceramides remain unknown. However, ceramide accumulation leads to ER stress which plays a key role in lipotoxic induced apoptosis [40,63,64]. Biden and co-workers showed that localized ceramide production and the associated decrease of SM levels in the ER in response to lipotoxicity is important to initiate ER stress in β cell [40,63]. Ceramide induced pancreatic β cell apoptosis is mediated by mitochondrial dysfunction and ROS production [60,61]. This result is also supported by the observation that ceramide dependent ROS production induced by gluco-lipotoxicity inhibits insulin secretion [8]. Evidence to date suggests that ceramide induce apoptosis through mitochondria injuries and subsequent production of ROS [65]. Nevertheless, the role of ROS in gluco-lipotoxicity is still unclear because in some cases, ROS have been excluded from ceramide-induced loss of function or apoptosis of pancreatic β cells [40,66]. Moreover, other hypotheses have been proposed about cellular signals involved in apoptosis of pancreatic β cell induced by ceramides. For example, it has been proposed that β cell apoptosis is induced through the activation of PP2A and the subsequent dephosphorylation of AKT [67], as seen in muscle tissues [68].

### 5.3. Role of Different Ceramide Species in Gluco-Lipotoxicity Induced Pancreatic β Cell Apoptosis

Ceramide species are distinguishable by the length and/or saturation of their *N*-acyl chains [47,69,70]. Because ceramide species have many biological roles depending on the cell type and on the inducing signals for their production [47], it is therefore important to determine changes in ceramide species in response to biological stimuli. Indeed, recent studies have reported distinct cellular functions for ceramides with specific *N*-acyl chain lengths [47,70]. The emergence of lipidomic analysis in the field of sphingolipid metabolism allows us to determine the effect of various treatments, not only on ceramide accumulation but also on changes in levels of ceramide species within the ceramide pool [52]. In pancreatic β cells, it appears that gluco-lipotoxicity modifies the levels of specific ceramide species, specifically C18:0, C22:0 and C24:1 [39]. In mammals, the variety of ceramide species relies on the existence of a family of enzymes, the ceramide synthases (CerS) [47,70]. Six CerS have been identified and they possessed a characteristic substrate preference for particular fatty acyl-CoA [47,70]. Interestingly, this increase in ceramide species is associated with an augmentation of CerS4 expression, which was found to play a critical role in pancreatic β cell apoptosis [39]. Collectively, the results suggest that gluco-lipotoxicity induces β cell apoptosis not only through the induction of *de novo* ceramide biosynthesis, but also through the formation of ceramides with specific carbon chain lengths rather than an overall increase in ceramide content. This hypothesis is supported by a recent study showing that pancreatic β cell apoptosis is potentiated when high glucose levels increased expression of FA elongase 6 (Elovl6) [71], an enzyme which catalyzes FFA elongation [72]. Importantly, the activity of Elovl-6 is mostly involved in elongation of *de novo*-synthesized palmitate to produce stearate, suggesting that gluco-lipotoxicity could mediate β-cell apoptosis at least through ceramide C18:0 by a concomitant up-regulation of CerS4 and Elovl-6. Recently, Prentki and co-workers have shown that gluco-lipotoxicity induced early changes in lipid partitioning in order to induce β-cell dysfunction and apoptosis [73]. Indeed, gluco-lipotoxicity induced the expression of proteins which favored FFA esterification, such as SCD-1 through FFA desaturation, and decreased the expression of enzymes involved in lipid oxidation, such as the β subunit of AMP-kinase. These results suggest that in addition to downstream lipid partitioning induced by gluco-lipotoxicity, the synthesis of specific ceramide species could also play a critical role in β cell apoptosis. However, the precise role of specific ceramide species in β cell dysfunction induced by gluco-lipotoxicity remains to be clarified. Indeed, a recent study showed that the inability of CerS2 knock-out mice to synthesize C22–C24 ceramide species did not result in defective insulin secretion [74]. Therefore, it will be important, in the future, to validate these observations *in vivo* by using rodent models of T2D associated to obesity, and ultimately in human islets of Langerhans in order to identify potent therapeutic targets to limit pancreatic β cell gluco-lipotoxicity.

## 6. Role of Sphingoid Base Phosphates in T2D

During the past decade, other sphingolipid metabolites, namely sphingoid base phosphates, have been shown to have a role in T2D. The sphingoid base phosphates are S1P and its analogue dihydroS1P, which are respectively produced by phosphorylation of Sph and DH-Sph by sphingosine kinase 1 (SphK1) and sphingosine kinase 2 (SphK2) [45]. S1P is known to induce proliferation, survival, migration and calcium mobilization in many cell types [42]. In T2D patients and rodent models of T2D, the activity of SphK1 and/or mRNA of SphK1 are increased in myocardium [75]. Interestingly, palmitate [76] or glucose [77,78], taken separately, not only increased sphingoid base phosphate levels but also SphK1 activity in different cell types such as muscle and endothelial cells. Moreover, treatment with S1P analogs protected cells from apoptosis [79,80] induced by hyperglycemia. In addition, *in vivo* over-expression of SphK1 gene in KK/Ay type 2 diabetic mice [81] or treatment mice under high fat diet with S1P analog [82] reduced the deleterious appearance of insulin resistance and T2D. Nevertheless, the role of the SphK1/S1P axis in T2D is still unclear. Indeed, induction of diabetes by an injection of streptozotocin leads to the activation of SphK1 associated to an inflammatory phenotype in vascular endothelium and retina [77,83]. Moreover, the SphK1/S1P axis appears to stimulate the expression of cytokine by adipocytes isolated from ob/ob mice [84] and in obese T2D patients [85].

### 6.1. Sphingoid Base Phosphates and Insulin Secretion

In pancreatic β cells, SphK1 and SphK2 and four of the five S1PR are expressed [85,86,87]. Expression and activity of SphK1 and SphK2 are stimulated by cytokines such as IL1β, TNFα and interferon-γ, but also by glucose and acetylcholine [77,87]. The role of S1P in insulin secretion is still unclear. Indeed, it has been shown that adding S1P to culture medium of pancreatic INS-1 β cell line and islets of Langerhans inhibits insulin secretion in response to glucose potentiated by GLP-1. The S1P1 receptor, coupled to Gi appears to be involved in this phenomenon [86]. In contrast to this study, S1P was found to stimulate secretion of insulin in islet of Langerhans [88] and in pancreatic HIT-T 15 β cell line [89,90]. Interestingly, Ozcan and colleagues showed that SphK2 plays a critical role in S1P production and insulin secretion induced by glucose [90]. To date, SphK1 has not been implicated in the control of insulin secretion. However, most of these studies have been conducted in pancreatic β cell lines or isolated normal islets. It will thus be important to investigate the *in vivo* role of sphingoid base phosphates by analyzing insulin secretion in available knock-out mice for SphK1 and SphK2 [91,92].

### 6.2. Sphingoid Base Phosphates Pancreatic β Cell Survival

In pancreatic β cells, the SphK1/S1P axis plays a critical role in survival. Indeed, adding S1P to culture medium of human or murine islets of Langerhans inhibited apoptosis induced by cytokines [93]. In this case, the anti-apoptotic effect of S1P is mediated by the S1P2 or S1P3 receptors, and the activation of the PKC pathway [93,94]. Interestingly, activation of SphK by glucose has also been implicated in β cell proliferation [76]. It was observed that gluco-lipotoxicity increased sphingoid base phosphates via up-regulation of the SphK1 pathway in pancreatic β cells [88]. Pharmacological inhibition of SphK activity drastically potentiates β cell apoptosis whereas over-expression of SphK1 partially inhibits β cell apoptosis induced by gluco-lipotoxicity. The anti-apoptotic effect of sphingoid base-1-phosphates are likely to be mediated by opposing signaling pathways to ceramide-induced apoptosis. Indeed, over-expression of SphK1 not only inhibited lipotoxicity-induced loss of mitochondrial transmembrane potential and cytochrome c release in pancreatic β cells [95] but also impaired protein trafficking between ER and Golgi which contributed to lipotoxicity-induced ER stress [88]. Another anti-apoptotic effect of sphingoid base phosphates involved the alteration of lipotoxicity-induced ceramide synthesis in pancreatic β cells [88]. While the mechanisms for this latter process remain to be established, it could potentially involve inhibition of CerS by S1P [96]. The *in vivo* protective role of SphK1 against pancreatic β cell apoptosis induced by gluco-lipotoxicity was supported by a recent study by Qi and co-workers who showed that while a high fat diet-fed wild-type (WT) mice developed glucose intolerance, all high fat diet-fed Sphk1 knock-out mice manifested evident diabetes, a phenomenon due to a drastic decrease in pancreatic β cell mass [95]. Nevertheless, the *in vivo* role of SphK1 still remains unclear since a recent study showed that under high fat diet-fed mice, SphK1 deficiency was associated with enhanced insulin signaling in adipose and muscle tissues and improved systemic insulin sensitivity and glucose tolerance [85]. Despite this recent study, the anti-apoptotic role of sphingoid base phosphates in pancreatic β cells are in agreement with the work of Boslem *et al*., who showed that ceramide removal from the ER, by favoring their conversion into glucosylceramides [40], is critical for β cell survival under gluco-lipotoxic conditions. Our findings suggest that the SphK1/sphingoid base phosphates axis could constitute a new therapeutic strategy for preventing pancreatic β cell death and thus the onset of T2D associated with obesity. In the future, it will be important to identify potent activators of the SphK1/sphingoid base phosphates axis in pancreatic β cells. In support of this, a recent work by Holland *et al*. [97] showed that adiponectin can preserve pancreatic β cell function by shifting the ceramide/S1P ratio towards higher relative levels of S1P.

## 7. Conclusions

**Figure 2:**
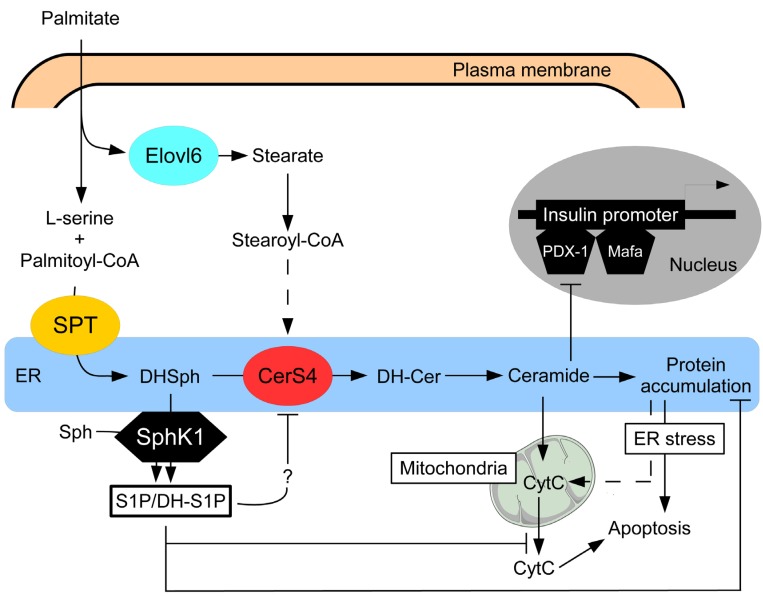
Role of sphingolipid biostat in β-cell apoptosis during gluco-lipotoxicity. Palmitate has been shown to induce ceramide accumulation by a dual mechanism involving serine palmitoyl-transferase (SPT) and the formation of ceramides with specific *N*-acyl chain lengths by ceramide synthase 4 (CerS4). Fatty acid elongase 6 (Elovl-6) provides preferential substrate for CerS4 by converting palmitate into stearate. Ceramides reduces insulin expression through inhibiting the binding of pancreatic and duodenal homeobox 1 (PDX-1) and Mafa transcriptional factors to insulin promoter. Ceramide accumulation can also induce pancreatic β cell apoptosis. However, palmitate also induces sphingosine kinase 1 (SphK1) expression, which will channel the preferential metabolic flow of newly formed dihydrosphingosine (DHSph) towards its phosphorylation into dihydrosphingosine-1-phosphate (DHS1P) and increase S1P levels. Accumulation of these sphingoid base phosphates in the ER will play a protective role against palmitate-induced ceramide-dependent apoptotic β cell death. Sphingoid base phosphates inhibit ceramide synthesis by acting probably on CerS4 activity and restore protein trafficking in the endoplasmic reticulum (ER), alleviating ER stress and attenuating cytochrome C (CytC) release from mitochondria. DH-Cer: dihydro-ceramides; Sph: sphingosine.

Because of their inter-convertibility and opposing effects, the dynamic balance between sphingoid base phosphates and ceramides has been proposed to be an important factor that determines cell fate [42], a concept called the sphingolipid biostat. The results of recent *in vitro* and in some cases *in vivo* studies lend credence to the observation that pancreatic β cell apoptosis induced by gluco-lipotoxicity is regulated by such a sphingolipid biostat (Figure 2). The existence of this biostat offers the possibility to find new therapeutic targets in order to limit pancreatic β cells failure observed during obesity, and therefore to prevent the appearance of T2D.

## Abbreviations

FFA: free fatty acid
LC-CoA: long chain acyl-CoA
S1P: shingosine-1-phosphate
CPT-1: carnitine palmitoyl-CoA transferase 1
ACC: acetyl-CoA carboxylase
FAS: fatty acid synthase
ER: endoplasmic reticulum
SPT: serine palmitoyl-transferase
DH-Sph: dihydrosphingosine
CerS: ceramide synthases
PASK: serine/threonine Per-Arnt-Sim domain-containing kinase
SMS1: sphingomyelin synthase 1
SphK: sphingosine kinase
